# Gene Tags Assessment by Comparative Genomics (GTACG): A User-Friendly Framework for Bacterial Comparative Genomics

**DOI:** 10.3389/fgene.2019.00725

**Published:** 2019-08-26

**Authors:** Caio Rafael do Nascimento Santiago, Renata de Almeida Barbosa Assis, Leandro Marcio Moreira, Luciano Antonio Digiampietri

**Affiliations:** ^1^Bioinformatics Graduate Program, University of Sao Paulo, Sao Paulo, Brazil; ^2^Adventist University of Sao Paulo, Sao Paulo, Brazil; ^3^Biotecnology Graduate Program, Núcleo de Pesquisas em Ciências Biológicas, Federal University of Ouro Preto, Ouro Preto, Brazil; ^4^Department of Biological Sciences, Federal University of Ouro Preto, Ouro Preto, Brazil; ^5^School of Arts, Science, and Humanities, University of Sao Paulo, Sao Paulo, Brazil

**Keywords:** user-friendly tools, systems biology, comparative genomics, orthologs, gene families

## Abstract

Genomics research has produced an exponential amount of data. However, the genetic knowledge pertaining to certain phenotypic characteristics is lacking. Also, a considerable part of these genomes have coding sequences (CDSs) with unknown functions, posing additional challenges to researchers. Phylogenetically close microorganisms share much of their CDSs, and certain phenotypes unique to a set of microorganisms may be the result of the genes found exclusively in those microorganisms. This study presents the GTACG framework, an easy-to-use tool for identifying in the subgroups of bacterial genomes whose microorganisms have common phenotypic characteristics, to find data that differentiates them from other associated genomes in a simple and fast way. The GTACG analysis is based on the formation of homologous CDS clusters from local alignments. The front-end is easy to use, and the installation packages have been developed to enable users lacking knowledge of programming languages or bioinformatics analyze high-throughput data using the tool. The validation of the GTACG framework has been carried out based on a case report involving a set of 161 genomes from the Xanthomonadaceae family, in which 19 families of orthologous proteins were found in 90% of the plant-associated genomes, allowing the identification of the proteins potentially associated with adaptation and virulence in plant tissue. The results show the potential use of GTACG in the search for new targets for molecular studies, and GTACG can be used as a research tool by biologists who lack advanced knowledge in the use of computational tools for bacterial comparative genomics.

## Introduction

Systems biology seeks to study the interaction between the components of a biological system holistically, mediated by several analytical tools, aiming the search for information capable of supporting the discovery of phenomena or complex biological processes (Chuang et al., 2010). Over the past years, such approaches, which have always developed from a multidisciplinary perspective, have made possible great discoveries involving new biomarkers of selection and diseases, targets for drug development, among others, all concurrently with the development of the robust platforms and computational tools for analyzing high-throughput data (Berg, 2014).

Despite the advances mentioned above, some challenges still exist. Among these, the search for specific genes that may be associated with certain phenotypes stands out. Such a search is a non-trivial task because it consists of solving a multifactorial problem (Casadesús and Low, 2006). In microbiology, this challenge is even more pronounced, as the functional characteristics of a gene may be directly associated with the biological processes of biotechnological interest or that allow a better understanding of the host’s immune response in the case of pathogenic microorganisms (Zamioudis and Pieterse, 2012; Campbell et al., 2017).

The development of new sequencing platforms in association with the set of “omics” sciences that seek to functionally analyze sequenced genes and genomes has substantially increased the volume of biological data available over the past years (Field et al., 2009). However, the understanding of genes’ specific functions has advanced modestly, despite the efforts of the scientific community (Chervitz et al., 2011; Berger et al., 2013). This is justified by numerous factors that hinder gaining such understanding. Some of them are inherent to the limitations and constraints of molecular techniques (Tierney and Lamour, 2005). However, some of them arise from two factors: 1) the lack of robust data analysis tools for different biological questions, many of which are specific to a particular type of biological knowledge, or 2) the existence of data analysis tools that make interpreting the processing mechanism or displaying the results generated by such tools challenging (Hillmer, 2015).

To make experimental validation more assertive, scientists from different fields have developed computational tools that allow integrating biological data using complex algorithms and enabling user interaction through user-friendly interfaces. It is in such a context that the need for user-friendly tools applied to systems biology arises, developed with an intuitive interface that allows biologist users to perform complex analyses, guiding them to answer biological questions.

In this study, we present a new user-friendly tool named Gene-Tag Assessment by Comparative Genomics (GTACG) applied to genetics or systems biology and developed for the comparative analysis of bacterial genomes, aiming the selection of genes for studying correlation of presence or absence of genes with lifestyle, virulence, among other biological questions.

GTACG allows interactive analysis and data visualization, always considering the comparison of phenotypic groups. Different characteristics are considered in this process, such as the composition of gene families as well as their individual alignments and phylogeny, producing more robust data than binary metrics. The result of the execution pipeline is a static website, which allows gaining easy-to-share data and specific results through URLs.

GTACG produces phylogenies based on different characteristics, which allows for a more detailed analysis of phylogenetic relationships, particularly when phylogenetically closely related organisms are being analyzed. Also, the framework presents a methodology for the discovery of genetic characteristics highly related to phenotypic characteristics in pangenomes. The genomes from the previous manual annotation are divided into groups to identify characteristics unique or more related to a particular group of interest. These characteristics have the potential to explain the different phenotypes among the genomes and may be the key for different kinds of research, such as the identification of biotechnological targets for disease control, the development of vaccines, among others.

The validation of GTACG’s functionality is established from the following biological question: is it possible to identify the potential genes that would justify the fact that some bacteria have the ability to survive in association with plants while others do not have such an adaptive characteristic? To answer this complex question, we analyzed a set of 161 genomes from the Xanthomonadaceae family using GTACG. This family is considered for analysis because it comprises genera of strictly phytopathogenic bacteria as well as those with distinct lifestyles not associated with plants. After the processing and presentation of the results, GTACG has proven efficient in answering the established question, allowing the identification of the potential gene families for the molecular studies of the plant–pathogen interaction in pathosystems of agricultural interest. In conclusion, therefore, GTACG can be used to answer similar questions at different levels of complexity, using any set of genomes previously established by users.

## Materials and Methods

**Figure 1:**
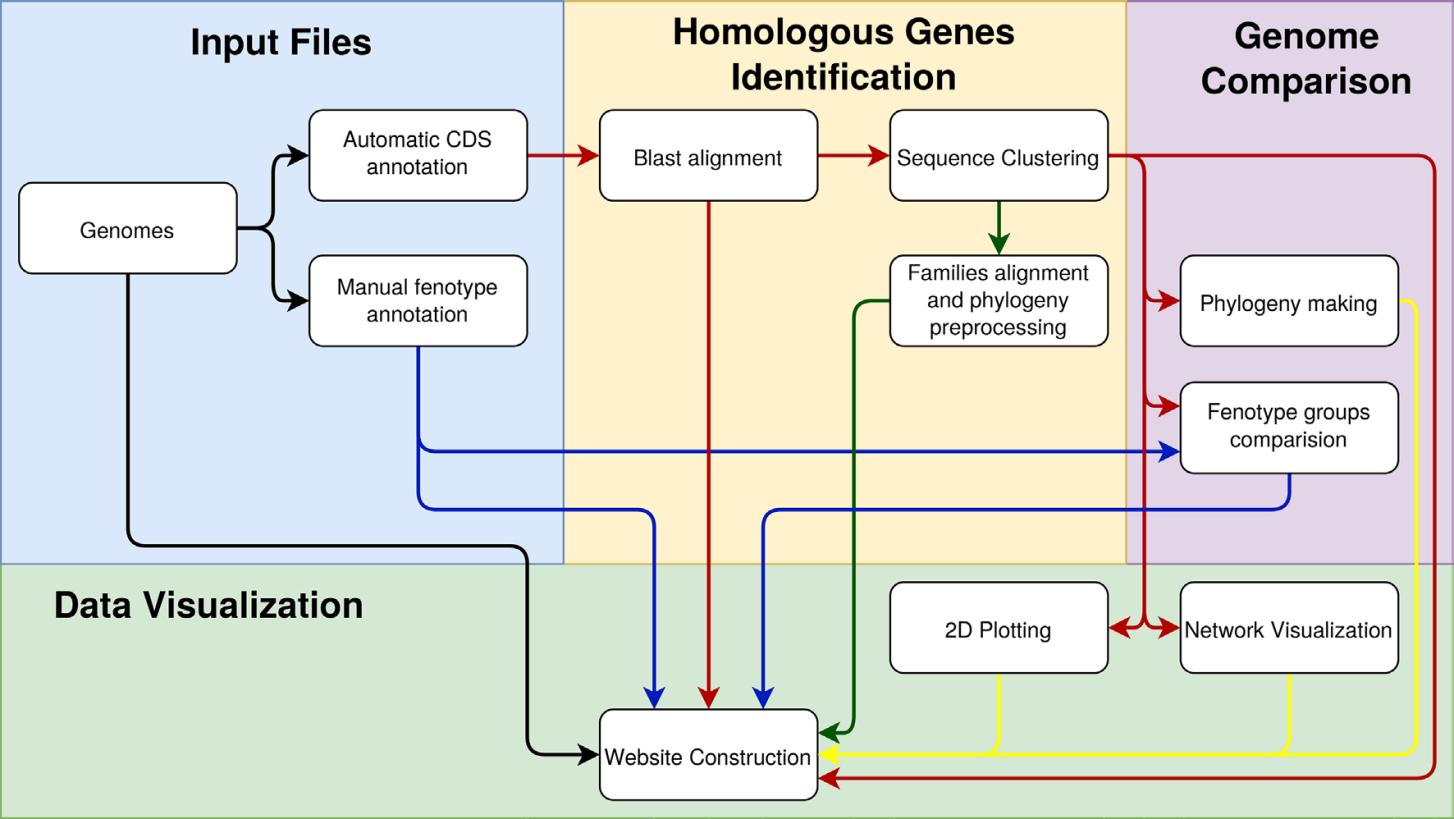
Steps involved in constructing the GTACG pipeline separated into three stages of pre-processing: the identification of homologous genes, the comparison of genomes, and the visualization of data. To facilitate the visualization of the relationships that the data have in each of the activities, the arrows were colored as follows: in black is the general data on genomes; in blue is the data about groups of genomes; in red is the data on the sequences; in yellow are the graphical results for visualization.

The environment as a whole can be divided into back-end and front-end. The back-end is developed in Java, which is the stage when the preprocessing of the genomic data provided by users occurs. Users provide data such as the complete genome sequence (in the FASTA format and multiple files if necessary), manual annotation of these genomes (in plain text files), and annotation of CDSs (preferably automatic annotation of sequences in the formats FASTA, gb, gbf, and gff). The GTACG execution pipeline is schematically described in **Figure 1** and has three main pillars: 1) identification of homologous genes, 2) comparison of complete genomes, and 3) genome visualization. In order to avoid inconsistencies between the annotations of the different genomes, all the genomes used were automatically reannotated using a RASTtk-based tool available at the PATRIC web service (Wattam et al., 2016).

### Identification of Homologous Genes

The first step is to calculate the local alignments of all CDSs against all CDSs using blastp to obtain the alignment length and E-value metrics. Then, a threshold of a minimum size of alignment associated with the degree of separability of the families is set by users. The E-value is automatically chosen to maximize the clustering coefficient of the graph which represented the relationships among CDSs and, therefore, maximizing the transitivity of the homologous correlations (Santiago et al., 2018). The result of this process generates layers of thresholds that indicates the decisions needed to identify the homologous gene families. These layers allow users to use different levels of trust to build gene families that can be chosen according to the goals of their research.

Also, two other steps were established for the subdivision of homologous CDS families. In the first step, a simple phylogenetic analysis is used, in which branches longer than a certain threshold are excluded, producing the division of a potentially homologous family into two or more orthologous families (Ding et al., 2017). Finally, a search for multidomain proteins is made, taking advantage of the asymmetry in the alignment graphs of each of the previously established families. A family with multidomain proteins, binding two or more CDS groups, is then subdivided into these groups. Unlike homology and orthology, this step resulted in intersecting subdivisions.

For each family, from the three depth levels (homology, orthology, and 102 domains), multiple alignments of the CDSs are done, and the generation of phylogenies is established using Clustal Omega (Sievers et al., 2011) and FastTree (Price et al., 2010), respectively. These data are preprocessed to generate a unified phylogeny, to calculate the metrics related to group phenotypes and for visualization in a graphical environment.

### Comparison of Complete Genomes

Using different approaches, three phylogenetic profiles are constructed from the families of homologous CDSs identified in the previous step. The first considers the presence or absence of each genome in the homologous gene families. From these data, a binary vector of characteristics is constructed, in which each characteristic represents a family and assumes the value 1 (one) if the respective genome has one or more CDS in the corresponding family and 0 (zero) otherwise. The junction of all these vectors is then presented to an algorithm for phylogeny inference. The second approach uses a distance matrix for phylogenetic inference constructed by the Euclidean distance between the binary vectors of characteristics. The third approach is based on the concept of supertree (Creevey and McInerney, 2004) and corresponds to a summary of the phylogenetic relationships among several taxa fed by a set of phylogenies. The set of phylogenies chosen is the set of phylogenies of each of the gene families (generated from the alignment of their sequences).

Regarding the investigation of genetic traits based on genome annotations, three categories of characteristics of the families are considered. However, most of the approaches comprised finding characteristics that are common to a certain group of genomes (genomes that share some characteristic of interest set up by users) and simultaneously uncommon to the others. For this investigation, the following categories of characteristics are considered: 1) The conformation of families, defined by families (individually or in combination) unique to a particular group of genomes or families more considerably present in a particular group of genomes. In this way, metrics are presented to indicate how many CDSs are present in the family that belongs to the genomes of a given group. This data is also presented in percentages, indicating how much these CDSs are representative of the total family size and how many genomes of the group are represented by the family. 2) The alignment of the sequences of the families, identifying specific amino acids variations more common to a certain group of genomes. To express this concept numerically, we developed a metric of dissimilarity that assigns a correlation weight to a given group for each base. 3) The phylogeny of the families, analyzing the grouping or separation of a certain group of genomes in the phylogeny in relation to the others. The Most Isolated SubTree (MIST) metric was developed to express this concept that shows the size of the largest subtree found of the phylogeny that has sequences only related to the group under analysis.

### Genome Visualization

Similar to the comparison of genomes, the visualization is also quite dependent on the conformation of the families. The homologous gene identification algorithm utilizes a graph-based algorithm, in which the sequences are represented as nodes and the alignments as edges. Given this data structure, the pangenome is then presented as a gene network, where each homologous family is represented as a connected component, providing a comprehensive notion of the pangenome situation. A force-directed algorithm (Kobourov, 2012) is applied to approximate or separate the sequences according to their edges.

A bidimensional mapping of the genomes is also performed using the same distance matrix constructed from the characteristic vectors described for the phylogeny construction. Using a Multidimensional Scaling algorithm (Borg and Groenen, 2005), the distance matrix is approximated to a bidimensional plane, proportionally preserving the distances in the plane from the distances present in the matrix, resulting in an overview of the proximity/distance between the analyzed genomes.

In this step, the data from all previous steps is consolidated in a static website, so it is unnecessary to use complex server configurations to take advantage of most system functions. This is justified by the fact that the system uses data produced by pre-processing. The website also does not require the installation of a database management system because the data is written as JavaScript scripts. Although the data is related to each other, these relationships are managed internally and not through a database, thus not requiring computational background by users, which makes the GTACG a typical user-friendly tool in genetic analysis.

The website format was chosen due to qualities such as the ease in publishing results, the flexibility to change the environment, and the reusability of the data in other programs or systems. On the other hand, it allows different filters on the data as well as the creation of different data groups, allowing a rich interaction and the visualization or analysis of only the information of interest set by users. Another advantage is the possibility of sharing, through URLs, pages, and search results, which makes the data generated accessible for collaboration between researchers.

### Case Studies: Validation of GTACG Functionality

To present the potentialities of this framework, we implemented a pilot study. The case study contains 161 genomes from the *Xanthomonadaceae* family, belonging to the genera *Pseudoxanthomonas* (3), *Stenotrophomonas* (19), *Xanthomonas* (125), and *Xylella* (14) (**Supplementary Table 1**). The choice was made because the first two genera are not associated with plants, while the latter two are strictly phytopathogenic (except one species), thus allowing the re-evaluation of the preliminary results pre-generated by our team (Assis et al., 2017).

## Results

Through a single package of compressed files containing source code and shell scripts, users can easily install all the tools to run GTACG on a Linux desktop or server. Once installed, users can load the genomes of interest, and automatically the GTACG will perform an automatic reannotation as a way to standardize the data to be compared.

The searches are flexible to meet users’ needs by providing several metrics that can be combined in a variety of ways and shared through URLs. The customization of all visualization data (alignments, phylogenies, and graphs) is also available, which can be exported in ready-to-publish formats such as SVG and high-quality PNG.

**Figure 2:**
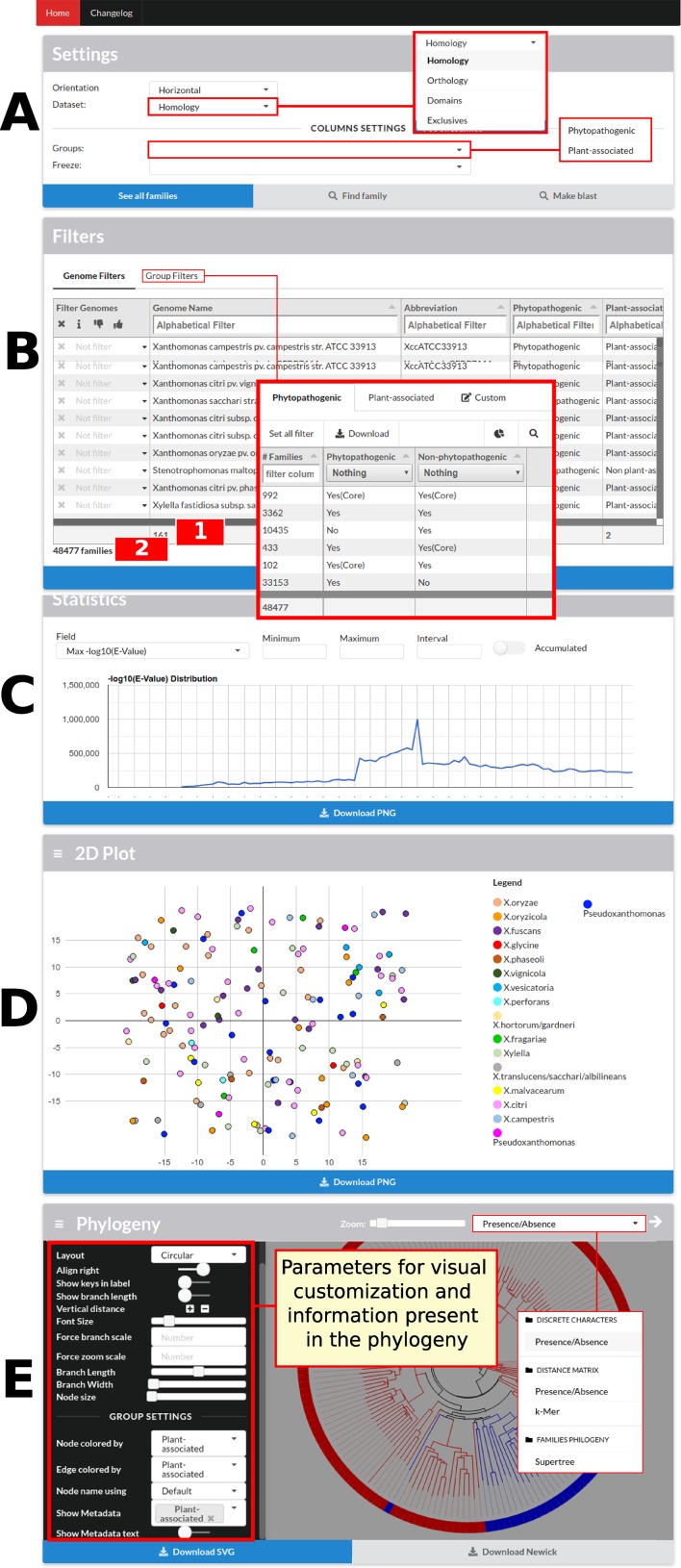
GTACG home screen. These results are divided into five sections: Settings, Filters, Statistics, 2D Plot, and Phylogeny. The first two sections are related to the subsequent family’s searches; the others are related to genome data. **(A)** The first allows the navigation between the different levels of clustering (homology, orthology, and domains). **(B)** The second allows filtering the presence/absence of the genomes or according to groups of genomes; this section also shows the number of genomes which are being filtered (label 1 in the figure) and the number of families after applying the filters (label 2 in the figure). **(C)** The third, Statistics, presents the graphs for the metrics related to families, sequences, and local alignments. **(D)** The fourth, 2D Plot, presents a bidimensional projection of the genomes. **(E)** Finally, Phylogeny presents the built phylogenies and customization options. Most sections fit users’ screen size.

The data visualization process has different levels of detail. In the initial screen of GTACG are the more macroscopic data that approach the visualization and interaction with genomes (**Figure 2**). In this screen, users can access the next level of detail regarding family’s search using basic settings in the Settings or Filters sections. In the second section, it is possible to define filters on the visualization of families in the results, based on genomes or groups. Families can be filtered on the basis of whether or not they require a particular genome to be present in the listed results, and information related to a particular genome can be ignored. It is also possible to easily find all the families that are shared or not shared by a certain group. In the following section named Statistics, graphs are built through the Google Charts library based on the metrics related to families, sequences, and local alignments. Finally, sections 2D Plot and Phylogeny present the chosen methods for visualization of genomes. Moreover, these two sections can be customized based on the groups of annotated genomes, in addition to several additional configurations. The phylogenies presented in GTACG use the Phylocanvas library for visualization.

The next level of detail concerns families. At this level, families can be found through statistical data, the sequences that compose them, and their base pairs respectively, available through buttons in the Settings section on the home screen. Families’ statistical data contain metrics such as the number of genomes shared by a family, the number of sequences, sequence length distribution, annotated function, the metrics discussed above for groups of genomes, and others based on the graphs constructed for the identification of families, distribution of amino acids in the alignment, and data on phylogeny. The statistical data refers to the degree of subdivision chosen for the families (homology, orthology, and domains, previously discussed in the *Materials and Methods* section), which can be changed in the initial screen of the system. These data are also available for download in formats that can be used to construct phylogenies (a distance matrix, for example) or in the Roary output format (Page et al., 2015) making use of a wide range of functions for the analysis and visualization of data already developed. In the sequence data, families are found according to the metrics present in each of the sequences that compose them, such as their annotated function, length, or position in the genome. In case there is a minimum server configuration (the execution of a script written in Node.js), it is possible to find families by Blast search against all sequences of the pangenome, with filters and results that are already the characteristics of this tool. These approaches have been structured as dynamic tables built with the Tabulator library, so users have at their disposal dynamic and complex filters adapted to work with mathematical and logical expressions as well as data grouping functions.

**Figure 3:**
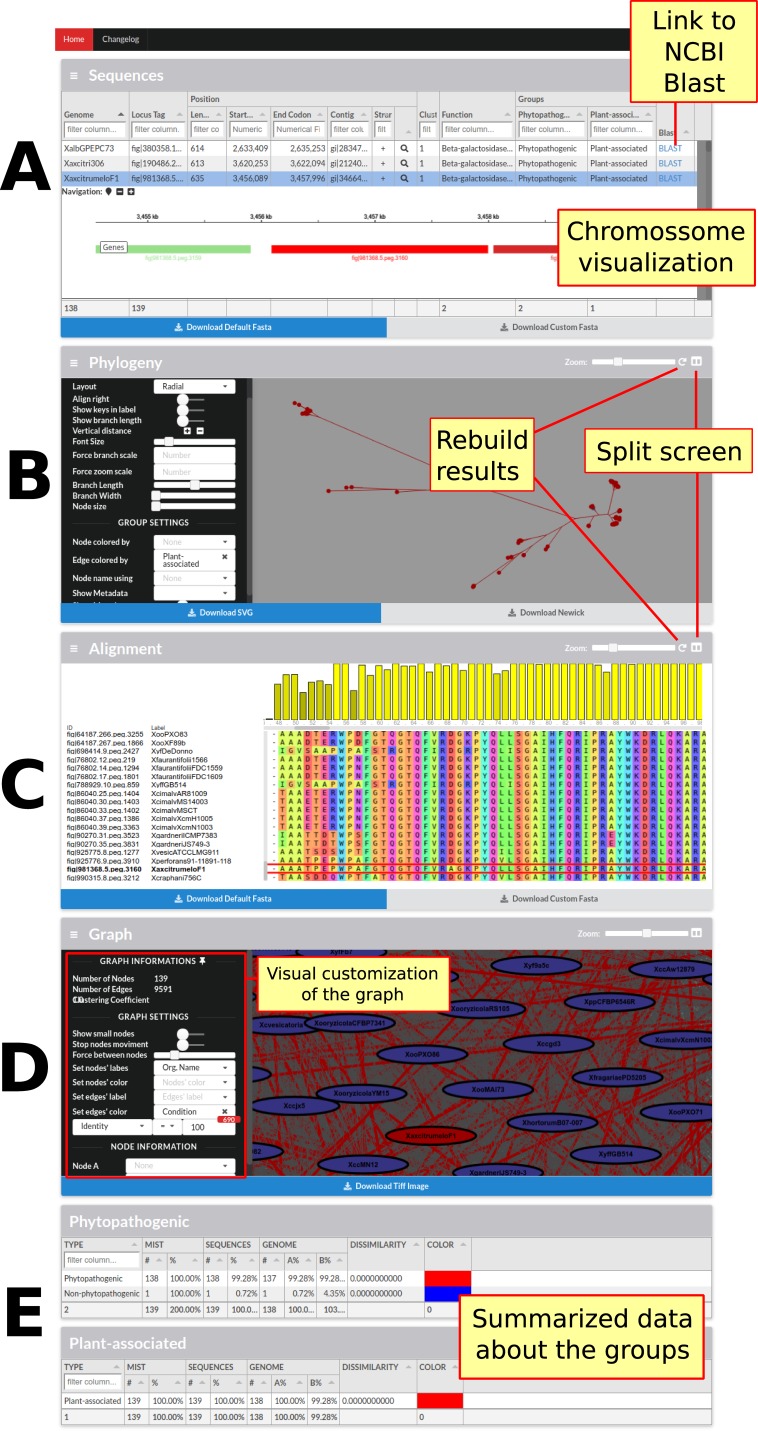
Screen containing the results of only one family. This screen contains four main sections followed by sections summarizing the family data in relation to the groups of genomes presented in the framework. **(A)** The first section has the sequence data and the data of their respective genomes; it is also possible to graphically visualize the position of each sequence in the genome as well as its vicinity. **(B)** In the next section, Phylogeny, it is possible to visualize, customize, and reconstruct (with different parameters) the phylogeny of the sequences. **(C)** The following section shows the alignment of all the sequences; it is possible to view, customize, and rebuild the alignment. **(D)** The fourth section presents the graph constructed to identify families, in which sequences are represented as vertices and local alignments as edges. The graph can be customized to highlight the alignments in accordance with some specific metrics. In this figure, the local alignments with identity equal to 100% are highlighted. **(E)** Finally, the last section summarizes the statistical data from each group of genomes with metrics about the number of genomes, dissimilarity, and MIST.”.

The last and lowest level of detail pertains to families. At this level, each family has its own page with its respective data (**Figure 3**). These pages have a total of five sections. In the first section, sequence data (annotation, length, among others) are combined with genome data (genome identification and annotated groups). Also, for each sequence, a link to the NCBI website to perform a Blast search is present. In case the server (a script written in Node.js) is configured, it is also possible to visualize the desired sequence and its synteny in the genome, due to the igv.js library. In the next two sections are phylogeny and sequence alignment respectively, using the Phylocanvas and MSAViewer (Yachdav et al., 2016) tools, and even when results are already pre-processed in the back-end, new results can be processed using FastTree (Price et al., 2010), PhyML (Guindon et al., 2010), RaxML (Stamatakis, 2014), Clustal Omega (Sievers et al., 2011) and MUSCLE (Edgar, 2004). The fourth section is devoted to the graph that generates the family, in the process of identifying families, representing the sequences as vertices and local alignments as edges. All this data is available for viewing and can be used to highlight edges by defining a condition, for example, highlighting the local alignments where the identity is less than 80%. Finally, the last section presents a statistical summary of the genome groups limited to family data.

Owing to all these possibilities, users are able to structure a research based on a top-down approach, first trimming with genomic data (such as phenotype annotation, phylogenetic data or exclusive genes statistics, for example) and then delving deeper to the point of better understanding the genetic mechanisms that can justify the initial data. The reverse is also possible, as users can find the orthologous family by having the amino acid sequence.

### The Case Study Validated by GTACG

The 161 genomes from the Xanthomonadaceae family employed in this study ranged in size from 2.5 to 5.5 million base pairs, with an average of 4,480 CDSs. The 743,920 CDSs were grouped into 48,477 homologous families, of which 4,287 were subdivided into 13,528 orthologous families, resulting in a total of 57,718 orthological families. This number of orthological families can be considered acceptable for this large and complex set of genomes. To obtain these results, two parameters were specified: 1) a maximum E-value threshold of 10-10 and 2) a minimum size of 45% for the alignments.

The main phenotype of interest evaluated in the proposal of GTACG validation is associated with the fact that some microorganisms from specific genera within the *Xanthomonadaceae* family have an adaptive association with plants, either as phytopathogens or not. It is important to emphasize that this characteristic was not mandatory for all the genomes investigated. This is justified by the fact that with this phenotypic characteristic, 139 genomes belonging to the genera *Xanthomonas* and *Xylella* and without this characteristic, 22 genomes belonging to the genera *Pseudoxanthomonas* and *Stenotrophomonas* were previously selected.

**Figure 4:**
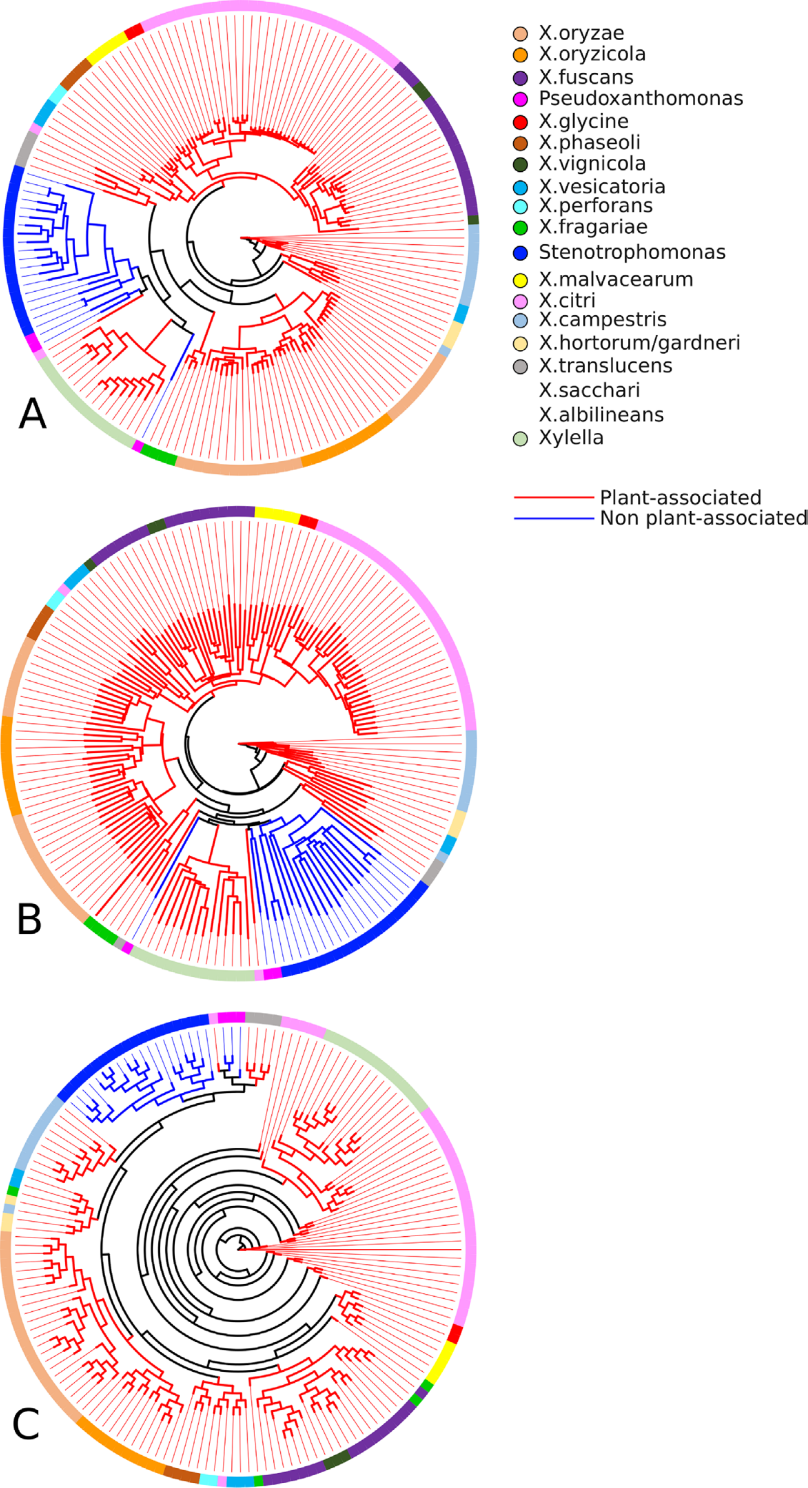
Phylogenetic profiles established by GTACG from the input genomes. The phylogeny **(A)** was inferred using the binary vectors for each genome; the positions of the vector represent the families and are defined as 0 or 1, depending on the presence/absence of the genome in the respective family; the method of inference was the parsimony program (pars) for binary features in the Phylip package. The phylogeny **(B)** was constructed using the distance matrix (using the Euclidian distance) of the binary vectors referred to above; the inference method chosen was the neighbor-joining also available in the Phylip package. The phylogeny **(C)** was constructed using a supertree that summarizes the collection of all the phylogeny constructed for the families; the tree of each family was obtained using the Clustal Omega to make the alignments and after that the FastTree produce the trees; the supertree method was the Quartet Fit algorithm with Nearest Neighbour Interchange available in the Clann.

As can be seen in **Figure 4**, the sets of associated and not associated with plants genomes are well separated from each other, which is reiterated in the literature (Sharma and Patil, 2011). In the tree constructed based on the binary vectors (**Figure 4A**) and in the tree constructed based on the distance matrix (**Figure 4B**), it is possible to clearly observe the separation of non-plant-associated microorganisms. Two exceptions can be observed in both trees, the clustering of *P. spadix* BD-a59 to plant-associated genomes and the clustering of *X. mangiferaeindicae* genomes into the cluster of non-plant-associated genomes. Moreover, the supertree (**Figure 4C**) presented a clustering with a more recent hypothetical ancestor for the non-plant-associated group, thus excluding *Xylella* (in discordance with the two phylogenies discussed above). This result corroborates with that of other studies that show that *Stenotrophomonas* is phylogenetically closer to *X. campestris* than to *Xylella* (Ramos et al., 2011; Naushad and Gupta, 2013).

No orthologous family presented the ideal behavior of being present in all genomes associated with plants and absent in all others. Nevertheless, very interesting results have been found that are consistent with the phylogenies constructed. It was found that 19 families of genes identified in 90% of the genomes associated with plants but were absent in genomes not associated with plants. Interestingly, the absent genomes are the same ones that were identified as separate groups in the phylogeny. In none of these 19 families, *X. mangiferaeindicae* is present. In three families, *X. albilineans* is also not present, and in two families, two strains of *X. translucens* and *X. sacchari* are also not present.

In another search, we also found nine families shared by all genomes associated with plants and less than 30% of the non-plant-associated genomes. Similarly, it should be noted that a few genomes not associated with plants have been integrated into this group and respective analysis. Interestingly, regarding these nine families, the number of non-plant-associated genomes that were included were very small (between three and six genomes). This result was partly expected, given the result presented by the supertree, as *P. spadix* BD-a59, *P. suwonensis* 11-1, and *P. suwonensis* J1 (genomes present in these families) were grouped in a branch with plant-associated genomes.

Also, nine protein families that compose the core genome have dissimilarity greater than 1% in their alignments, indicating amino acids with mutations more correlated to the genomes associated with plants. Finally, another 13 families from the core genome were isolated in a single branch of the phylogeny containing all sequences from microorganisms associated with plants.

## Discussion

### Pangenome Analysis Tools

The analysis of pangenome date back more than a decade (Vernikos et al., 2015). Several published works and computational tools are available, some of which using a similar approach presented in GTACG to study the genomes based on the clusters of homologous families (or orthologous). However, most of these works and tools are limited to global numerical analyses such as finding the different categorizations of the core genome or counting the number of unique genes in the analyzed genomes (Laing et al., 2010; Zhao et al., 2011; Benedict et al., 2014; Page et al., 2015; Zhao et al., 2018). Another common approach of these tools is the search for a reliable phylogeny from the raw input data, with the possibility of generating a rapid alignment of the genomes and not limited to the low resolution of some phylogenetic markers (Clarridge, 2004).

However, families of sequences or homologous genes have a wide range of information to be mined. It is in this context of a more refined search for information that the number of works and tools available still have limitations. Some of them, although discussing similar problems, use manual methods, which de-characterize them as potential user-friendly tools in systems biology.

Regarding the automatic methods already developed for the analysis of pangenome and homologous/orthologous genes or sequences search (some of them listed in **Table 1**), the PGAT (Brittnacher et al., 2011), the PanX (Ding et al., 2017), and the Obolski (Obolski et al., 2018) stand out. Although the PGAT provides a wide range of possibilities for gene searches with specific interests, it is limited, as it allows such search to be established only by a particular set of genomes. Moreover, one of the main limitations of the PGAT lies in the rigidity of not allowing approximate results to be found, a limitation also shared by BPGA (Chaudhari et al., 2016) that presents searches for phenotypic characteristics, but with inflexible search formats. For example, if any phenotype has not been correctly annotated (or expressed) by users, it will not be easily found, thus requiring many consecutive searches to solve the problem. Although the PGAT is able to present the results as a website, the specificities of the results (such as the result of a search) are not easily shared. PanX also presents the results in a website but more dynamically than PGAT. However, the search options are still limited to the basic statistical data on families such as the number of genomes present, and therefore there is a possibility of searches that support the study on phenotypes. An interesting advantage of the PanX is the visualization of family’s phylogenetic trees using metadata such as phenotypes from genomes as visual support. Finally, Obolski uses a Random Forest algorithm to find the families most correlated with the invasiveness phenotype, as presented by some strains of *Streptococcus pneumoniae*.

**Table 1:** Comparison of the main functionalities of some comparative genomics frameworks.

	GTACG	BPGA	PanX	PGAT	PanGP	PGAP	Panseq	ITEP	Get Homologues
Identification of phenotype-specific genes – list	X	X		X					X
Identification of phenotype-specific genes – metrics	X								
Distribution of core, accessory and unique genes	X	X	X						
Pangenome profile analysis	X	X			X	X			X
Size of core and pan-genome	X	X	X			X	X		X
Extraction of core, accessory and unique genes’ sequence	X	X						X	
Evolutionary analysis	X	X	X			X	X	X	X
Protein/gene clustering	X	X	X	X		X	X	X	X
Multilevel perspective of the genes	X		X	X				X	
Input data from user	X	X			X	X	X		X
Easy to share results	X			X			X		
Integration with roary scripts	X								
Data preparation	C	C	C	N/A	G	C	C	C	C
User interface	W	GO	W	W	GO	GO	GO	GO	GO
References		Chaudhari et al. (2016)	Ding et al. (2017)	Brittnacher et al. (2011)	Zhao et al. (2014)	Zhao et al. (2011)	Laing et al. (2010)	Benedict et al. (2014)	Contreras-Moreira and Vinuesa (2013)

Data preparation: C, Command line; G, Graphical interface.

User interface: W, Website; GO, Graphical output.

PanSeq Laing et al. (2010), as well as PanX and PGAT, also make the results easily available (*via* URLs), but as a service which provides only files with specific results, without customization and any interaction with the user. In general, the rest of the available frameworks are quite focused on an experience restricted to text commands, such as ITEP or get_homologues, or limited interactive interfaces, such as PGAP (Zhao et al., 2011) that has been recently extended with graphical interfaces (Zhao et al., 2018).

Based on the description of the qualities and limitations of the tools mentioned above, GTACG is able to combine the main advantages of all of them, besides having its own algorithm for the identification of homologous gene families with different levels of grouping, which minimizes some of the limitations imposed by other tools. Also, GTACG stands out by facilitating data presentation and the sharing of search results, a feature that is highly desirable in a user-friendly tool for systems biology. Although it does not cover all the diversity of software that address pangenome, owing to the existence of an open and easily modifiable environment, GTACG requires less effort to program new content, thus reducing the difficulties imposed by some tools aimed at the study of systems biology (Hillmer, 2015).

### The GTACG: Structural and Functional Characteristics

Some demands and difficulties imposed by the tools developed for studying systems biology guided the development of GTACG. GTACG was developed in consideration of the following:

#### Easy to Load the Information to be Analyzed

As it is aimed at the interdisciplinary public, the results were produced from files commonly used in genomic projects (for example, fasta, gb, and gff), easily obtained through NCBI and automatic annotation tools, and the interaction of the results with users occurs through a graphical environment. This allows users to load an unlimited number of genomes.

#### Minimizes the Propagation of Annotation Errors

Perhaps the most critical decision in a project on pangenomes concerns the formation of families of homologous sequences, especially if the problem is aggravated in situations where the sequence was annotated incorrectly (Devos and Valencia, 2001; Green and Karp, 2005). This leads to error propagation, and it is deterministic in the characterization of gene families incorrectly identified as homologous, thus creating false positive or false negative errors that are difficult to be identified. Therefore, the first step of GTCAG was established to standardize the CDSs’ annotation through an automatic annotation, as many genomes present in the NCBI database were submitted using different methodologies and at different times (Klimke et al., 2011).

#### Accuracy in the Clustering Method

Once the annotations have been standardized, another parameter crucial for the quality of the tool is the identification of the gene families, which many other studies have chosen to use—Markov Cluster Algorithm (MCL) and its derivatives (Enright et al., 2002; Li et al., 2003). However, this is a general-purpose clustering method. In this work, GTACG was chosen because it was developed with the implementation of the Multilayer Clustering, which is a more stringent parameter to be used in sequences from phylogenetically closer genomes. Also, this algorithm uses global decisions, considering the influence of all sequences on the formation of families, as the relationships between the sequences in pangenome studies are much more homogeneous than more diverse sequences.

#### Accuracy in the Search for Families of Sequences or Homologous Genes

The identification of homologous genes is a critical step. It impacts all obtained results such as phylogeny, searches for families, genome visualization, among others. To deal with this task, GTACG uses Multilayer Clustering (Santiago et al., 2018) instead of TribeMCL or OrthoMCL, which are more commonly used among known solutions. A detailed comparison of Multilayer Clustering and TribeMCL results considering a subset of 69 genomes from the 161 of the case study can be found in Santiago et al. (2018). These algorithms achieved comparable results when multidomain proteins are not considered. But, considering multidomain proteins, Multilayer Clustering achieved better results. Moreover, the impact of the decisions made by Multilayer Clustering is easier to understand, as the basic knowledge about alignment tools is enough to understand clustering decisions. It is opposite to MCL, which does not provide a transparent picture to users concerning what decisions impact homologous identification (Santiago et al., 2018).

#### Dynamic and Easy-to-Use Graphic Interface

All the interface was developed together and intended for biologists. Acknowledging the interdisciplinary public, some concerns were considered. The first concern was to create an environment that do not need complex server configurations, allowing computer non-specialist users to publish their results. The second and more important concern was to develop a dynamic system and an easy-to-use interface. The interface was modeled as a website using common internet symbols and icons to facilitate user learning. The pages were divided into genomic information (and visualization), family pre-processed metrics, and individual family information, designed as a top-down approach. Finally, the last concern was to create customizable graphics to allow users to express their ideas better. Moreover, the graphics could be exported to ready-to-publish formats (SVG, high-quality PNG, and TIFF).

#### Support for a Lifecycle Research Project

Considering all the features mentioned above, GTACG presents the qualities to support the work of researchers in different steps of the lifecycle of a research project. In the first step, GTACG supports researchers to obtain genomes directly from the NCBI database and, in a row, automatically reannotate them. Also, the methodology of the identification of homologous genes is covered, providing comprehensive results of clustering through the Multilayer Clustering. In the analysis step, GTACG allows researchers to test plenty of hypotheses and find data that can conduce to new hypotheses, collaboratively through URLs. Finally, the same environment of analysis serves to turn the data public and generate graphics with enough quality to support scientific publication. Thus, GTACG is able to support the full lifecycle of pangenome research without requiring computing knowledge.

### Performance of the Pipeline Execution

GTACG presents fairly complete results covering different stages of pangenome research. In general, this process starts after the reannotation of the sequences and the production of local alignments, these steps are the most computationally costly.

The total time of the automatic annotation, as well as the quality and specificity of its results, is quite dependent on the choice of the tool used. This step is quite costly and some tools require a manual effort from the researchers. However, it is an inevitable step to minimize methodological errors in many pipelines of tools based on homologous gene identification.

In order to evaluate the performance of the subsequent steps, five datasets were prepared with 10, 20, 30, 40 and 50 *Xanthomonas* genomes. These genomes are presented in the **Supplementary Table 1**, and the execution times are present in the **Supplementary Table 2**.

**Figure 5:**
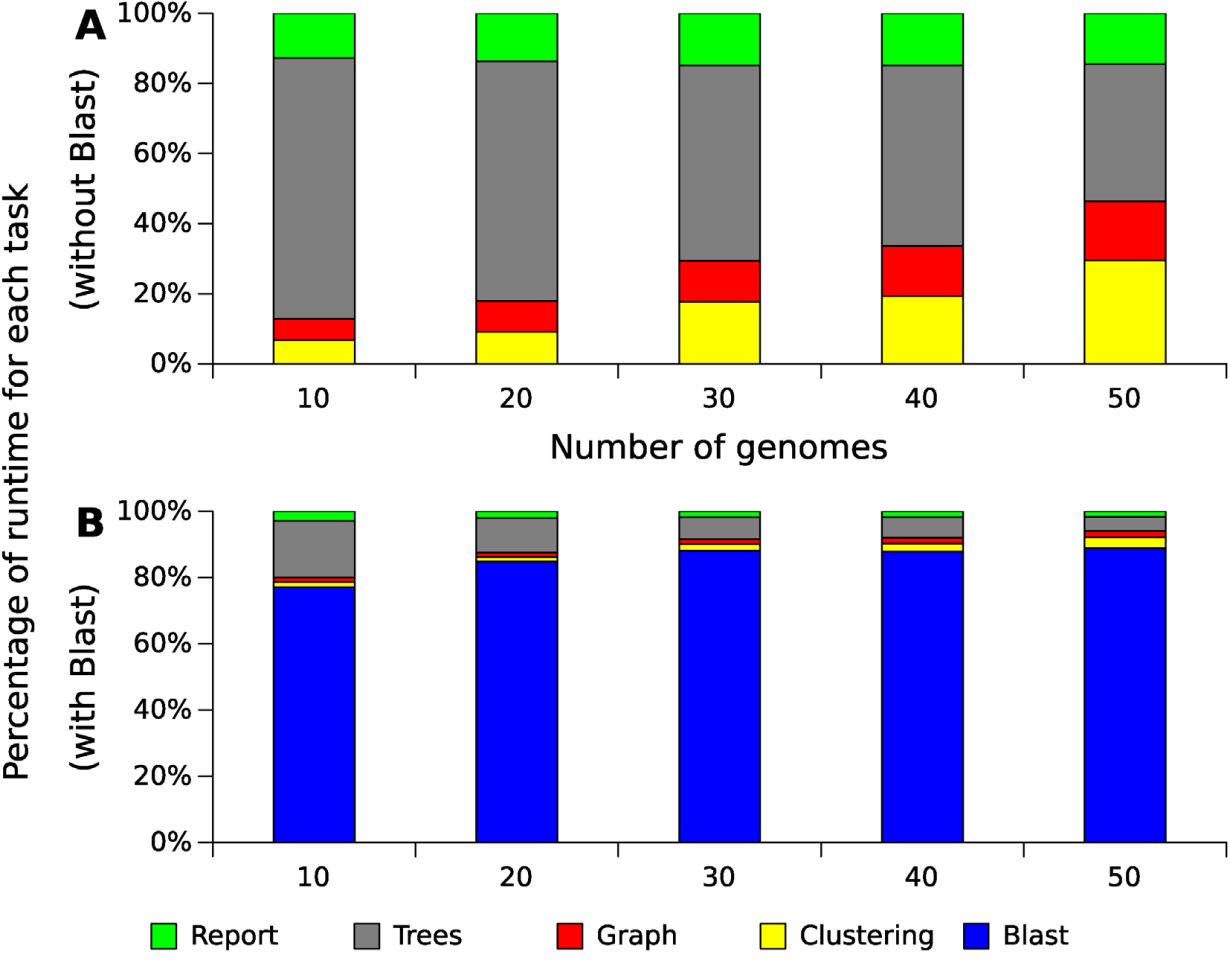
Relative runtime for GTACG’s main tasks with different datasets of Xanthomonas genomes. These results were obtained using a computer with an Intel(R) Xeon(R) CPU E5-2620. This computer has 24 cores, but only 20 of them were used. As Blast's alignments correspond to the majority of the consumed time, section **(A)** present the time spent excluding the time spent with Blast, while section **(B)** present the time including Blast.

**Figure 6:**
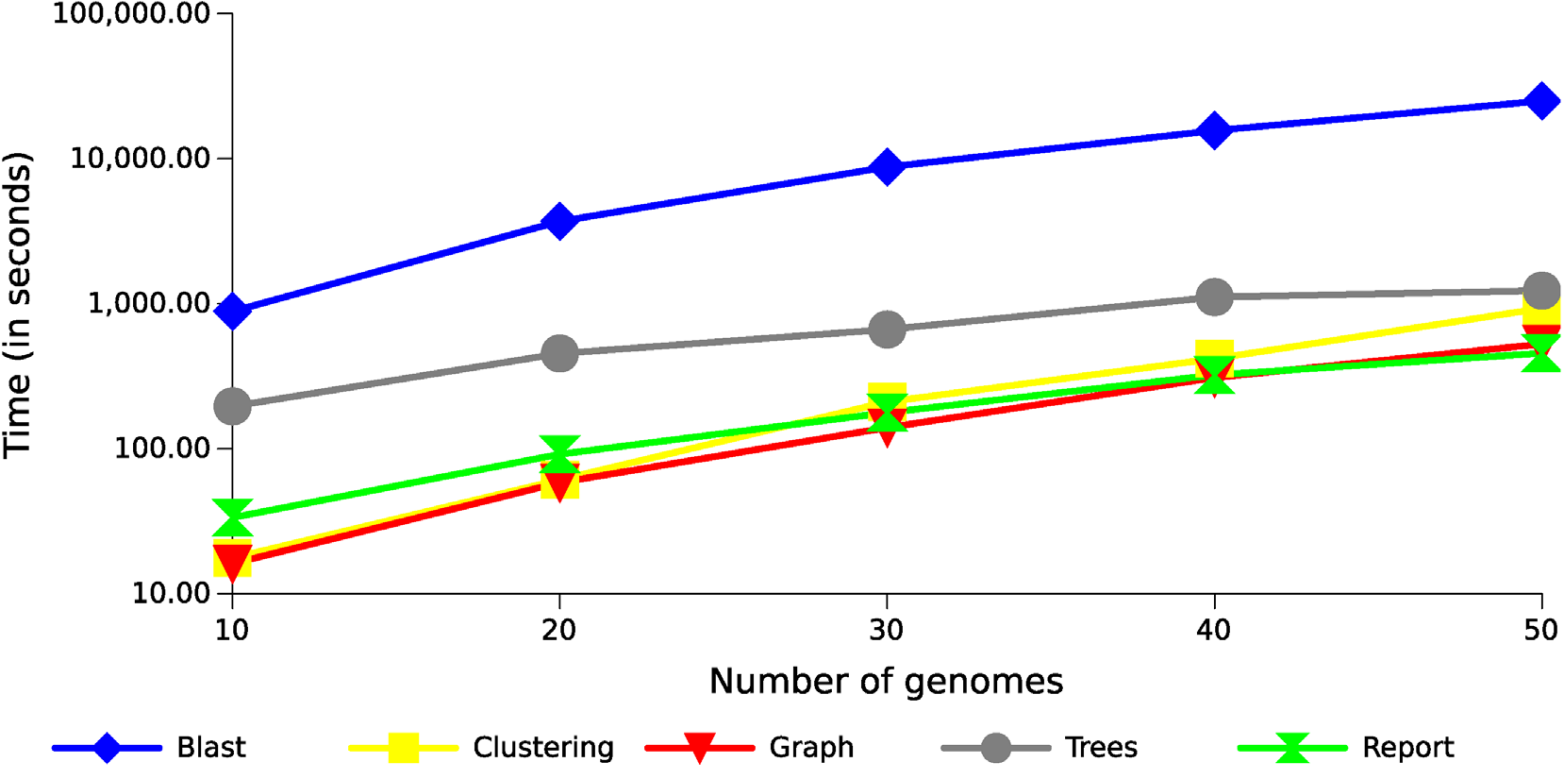
Runtime with different datasets of *Xanthomonas* genomes. The results show the execution time growth of the GTACG’s main tasks, according to the number of genomes in the datasets.

The step of producing local alignments of all sequences against all sequences was performed using BLAST (blastp), and is currently the most costly part of the whole process, consuming between 75% and 95% of the execution time for these datasets (**Figure 5** shows the result using 20 threads). Although this result can be accelerated through multithreading, the tendency of this consumption is exponential, as in the case presented in this figure, because the number of alignments produced increase exponentially with the increase of genomes. The remaining operations also tend to be exponential following the growth of the alignments (**Figure 6**). The most costly task after the alignments is the preparation of the multiple alignments and trees for each of the families, but this step follows a more linear trend.

A very promising alternative to the use of Blast is the MMseqs2 (Steinegger and Söding, 2017) with a sensitivity of 7.5, which considerably reduced the local alignment execution time (between 30 and 35 times), while maintaining similar results both in the tests datasets and in the case study discussed below.

Although GTACG takes longer to compute than other frameworks, such as Roary (Page et al., 2015), BPGA (Chaudhari et al., 2016) or PanGP (Zhao et al., 2014), GTACG provides more information for the users, different results and more tools to help the pangenome analysis in a simple and practical way for users with no programming skills.

### The Case Study Validated by GTACG

Considering the case study of 161 genomes from the Xanthomonadaceae family, all searches were done simply and efficiently, making the discovery of knowledge about phenotypes easier. Although these results are not sufficient to determine whether there is, in fact, the participation of which one of these families to express the phenotype, it is a starting point that can guide new laboratory studies.

**Figure 7:**
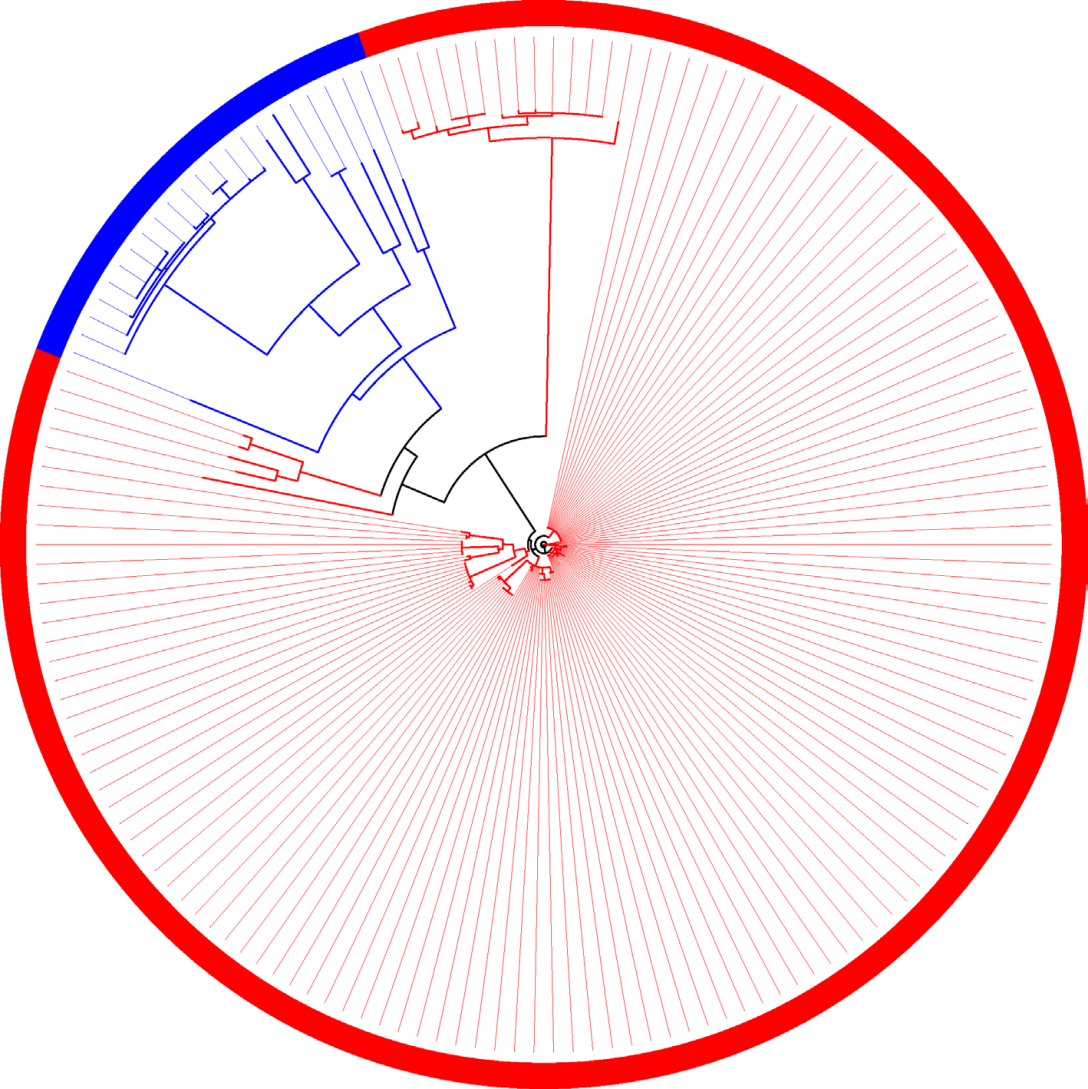
Family of orthologous sequences in which all sequences from plant-associated genomes are isolated from the other genomes.

The same behavior observed in the phylogeny is reflected in the composition of families (**Figure 7**). Even though the two groups (plant-associated and non-plant-associated) are well divided, there are branches involving few genomes in which the groups are mixed. There are 19 families unique to plant-associated genomes, and plant-associated genomes are present in at least 90% of them. *X. mangiferaeindicae* does not have genes in any of these families, and among 15 of them, it is the only one absent among plant-associated genomes. Of the four remaining families, one does not contain only *X. albilineans*, a microorganism vastly studied for being unique within this family and probably resulting from a process of genome reduction (Pieretti et al., 2009). In two other families, the same genomes grouped with non-plant-associated genomes, as described by the supertree, are absent. Considering these 19 families, most of them may be important for the metabolic interaction with plants, and therefore, *X. mangiferaeindicae* would have adapted to use an alternative strategy as well as *X. albilineans* could have adapted to using a reduced set of genes from these families. Finally, among this set of families, one of them do not contain any of the four strains of *X. fragariae* (besides *X. mangiferaeindicae*).

On the other hand, considering the families that comprise all the plant-associated genomes (but not exclusively them), there is a family that contains the same three non-plant-associated genomes grouped with the plant associated with the method of the supertree: *P. suwonensis* 11-1, *P. suwonensis* strain J1, and *P. spadix* BD-a59. Also, eight families contain, additionally to plant-associated genomes, genes from *S. nitritireducens*, *Stenotrophomonas* sp. KCTC 12332, and *S. acidaminiphila*. This can be explained by the hypothesis that perhaps the cited families are important to allow the association with plants, but some genomes potentially cannot express these genes and therefore would not express the phenotype either or the possibility that the genomes themselves were erroneously annotated.

Based on the alignments produced by the families, nine cases were found presenting amino acids with specific mutations in the plant-associated genomes with dissimilarity greater than 1%. The data below that indicates a 1% threshold does not yield very conclusive results, showing many non-exclusive mutations. Besides, from the phylogenies constructed based on the alignments, it was found that 19 families can be perfectly divided into both groups, as shown in **Figure 7**. By itself, this result does not imply that this is the most appropriate phylogeny to represent the evolution of the genomes, but as the phylogeny is an analysis derived from the combination of amino acids, this result indicates a significant difference observed by that amino-acid combination.

### Functional Description of Protein Families Found Exclusively in Plant-Associated Genomes

**Figure 8:**
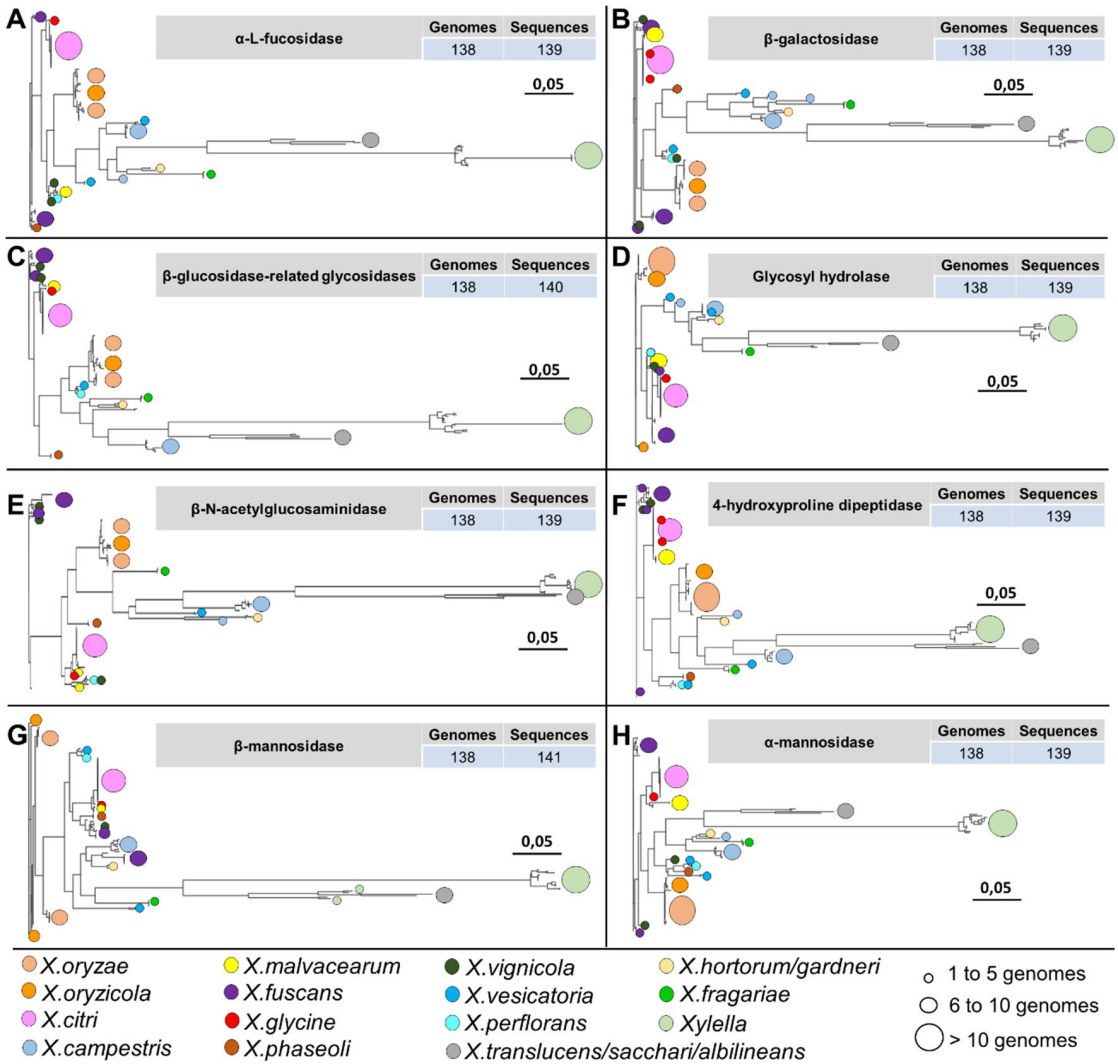
Phylogenetic analysis of 8 out of 19 protein families identified only among the genomes associated with the plants belonging to the family *Xanthomonadaceae*. The identification of circles, colors, and sizes is not provided by the tool; they have been inserted in this context only to facilitate the description of the identifiers. It is possible to observe a pattern in the topology of the phylogenies of the hydrolases, always with larger branches for organisms of the genus *Xylella* and *Xanthomonas translucens*, *X. sacchari*, and *X. albilineans*. **(A)** alpha-L-fucosidase family. **(B)** beta-galactosidase family. **(C)** beta-glucosidase-related glycosidases family. **(D)** glycosyl hydrolase family. **(E)** beta-N-acetylglucosaminidase family. **(F)** 4-hydroxyproline dipeptidase family. **(G)** beta-mannosidase family. **(H)** alpha-mannosidase family.

Among the 19 protein identified in at least 134 phytopathogen genomes in this study, eight protein families are involved in N-glycan degradation. Interestingly, all genes related to N-glycan degradation are located in the same genomic region constituting a cluster (nix) together with cutC (resistance to copper) and are responsible for the cleavage of the N-glycan in different glycosidic bounds (**Table 2** and **Figure 8**). Plant-pathogen interaction is driven by evolution of bacterial virulence proteins to induce virulence and modulate plant immune response alongside with evolution of plant proteins to recognize bacterial effectors and induce specialized immune response leading to resistance. Plant pattern-recognition receptors (PRR) are responsible for recognition of pathogen-associated molecular pathogens (PAMP) and activation of pathogen-triggered immunity (PTI). Häweker et al. (2010) showed that PRR require N-glycosylation to mediate plant immunity. By degrading the N-glycan associated with plant-receptors, the plant host is no longer able to recognize and activate the immune response, thus allowing greater success of colonization and adaptation of these bacteria within the host.

**Table 2:** Characterization of the 18 protein families exclusively identified in genomes of bacteria associated with plants.

Function	Gene name	Ref. Locus Tag	# Genomes	# Paralogs	Pathway	SP	Refs
Conserved hypothetical protein (putative lipase)	*lesA (lipA)*	XAC0501	134	27	Lipid metabolism	N	Aparna et al. (2009); Nascimento et al. (2016); Assis et al. (2017)
Peptidase M16 family/Zinc protease/Insulinase family protein	—	XAC0609	138	1	Peptidases	Y	Zhou et al., 2017
Low molecular weight heat shock protein/Molecular chaperone	*hspA*	XAC1151	138	1	Chaperones and folding catalysis	N	Lin et al. (2010)
Cytochrome O ubiquinol oxidase subunit IV	*cyoD*	XAC1261	138	2	Oxidative phosphoryla-tion	N	Lunak and Noel (2015)
Conserved hypothetical protein	—	XAC2544	137	2	Unknown function	Y	—
Predicted 4-hydroxyproline dipeptidase/Xaa-Pro aminopeptidase	*pepQ*	XAC2545	138	1	Metallo peptidases	N	—
Alpha-L-fucosidase	*nixE*	XAC3072	138	1	N-glycan metabolism	Y	Boulanger et al. (2014); Dupoiron et al. (2015); Assis et al. (2017)
Hypothetical protein (putative glycosyl-hydrolase)	*nixF*	XAC3073	138	1	N-glycan metabolism	Y	Boulanger et al. (2014); Dupoiron et al. (2015); Assis et al. (2017)
Beta-hexosaminidase/Beta-N-acetylglucosaminidase	*nixG*	XAC3074	138	1	N-glycan metabolism	Y	Boulanger et al. (2014); Dupoiron et al. (2015)
Beta-mannosidase	*nixH*	XAC3075	138	3	N-glycan metabolism	Y	Boulanger et al. (2014); Dupoiron et al. (2015)
Beta-glucosidase-related glycosidases/Gluca-beta-glucosidase	*nixI*	XAC3076	138	2	N-glycan metabolism	Y	Boulanger et al. (2014); Dupoiron et al. (2015); Assis et al. (2017)
Hypothetical protein (putative glycosyl-hydrolase)	*nixJ*	XAC3082	138	4	N-glycan metabolism	Y	Boulanger et al. (2014); Dupoiron et al. (2015)
Alpha-1,2-mannosidase	*nixK*	XAC3083	138	1	N-glycan metabolism	N	Boulanger et al. (2014); Dupoiron et al. (2015)
Beta-galactosidase	*nixL*	XAC3084	138	1	N-glycan metabolism	N	Boulanger et al. (2014); Dupoiron et al. (2015); Assis et al. (2017)
Cytoplasmic copper homeostasis protein CutC	*cutC*	XAC3091	138	2	Copper metabolism	N	—
3-isopropylmalate dehydrogenase/Isocitrate dehydrogenase	*leuB*	XAC3456	134	1	Leucine biosynthesis	N	Laia et al. (2009); Moreira et al. (2017)
Integral membrane protein	—	XAC4076	134	1	Unknown function	N	—
N-acetylglucosamine-regulated/TonB-dependent receptor	*nixD*	XAC4131/3071	138	10	TonB receptors/N-glycan metabolism	Y	Blanvillain et al. (2007)
Conserved hypothetical protein	—	XAC4164	137	1	Unknown function	Y	Jalan (2012)

SP, signal peptide; Y, yes; N, no.

Additionally, other proteins identified are involved in adaptation, including two peptidases [homologous to XAC0609 (Zhou et al., 2017) and PepQ-XAC2545] and three hypothetical proteins (homologous to XAC2544, XAC4076 and XAC4164) (**Table 2**). Analysis of the sequence of XAC0501 revealed that this gene coded by LesA/LipA is a key virulence factor required for *Xylella fastidiosa* pathogenesis in Grapevines (Nascimento et al., 2016), *Xanthomonas citri* in citrus (Assis et al., 2017) and *Xanthomonas oryzae* in rice (Aparna et al., 2009). The other four genes may be related with adaptation. HspA has been described as a chaperone very important as a protective agent during the storage of proteins in *Xanthomonas campestris* (Lin et al., 2010). CyoD coded by a cytochrome O ubiquinol oxidase subunit IV that is a component of the aerobic respiratory chain that predominates when cells are grown at high aeration (Lunak and Noel, 2015). LeuB coded by a 3-isopropylmalate dehydrogenase that was upregulated in *Xanthomonas axonopodis* pv. *citri* (Xac) 1, 3 and 5 days after inoculation (Moreira et al., 2017), and when mutated the absence of leuB showed reduction of Xac virulence in the compatible host (Laia et al., 2009). Only homologous to XAC4076 coded by an integral membrane protein was not investigated in other studies.

**Figure 9:**
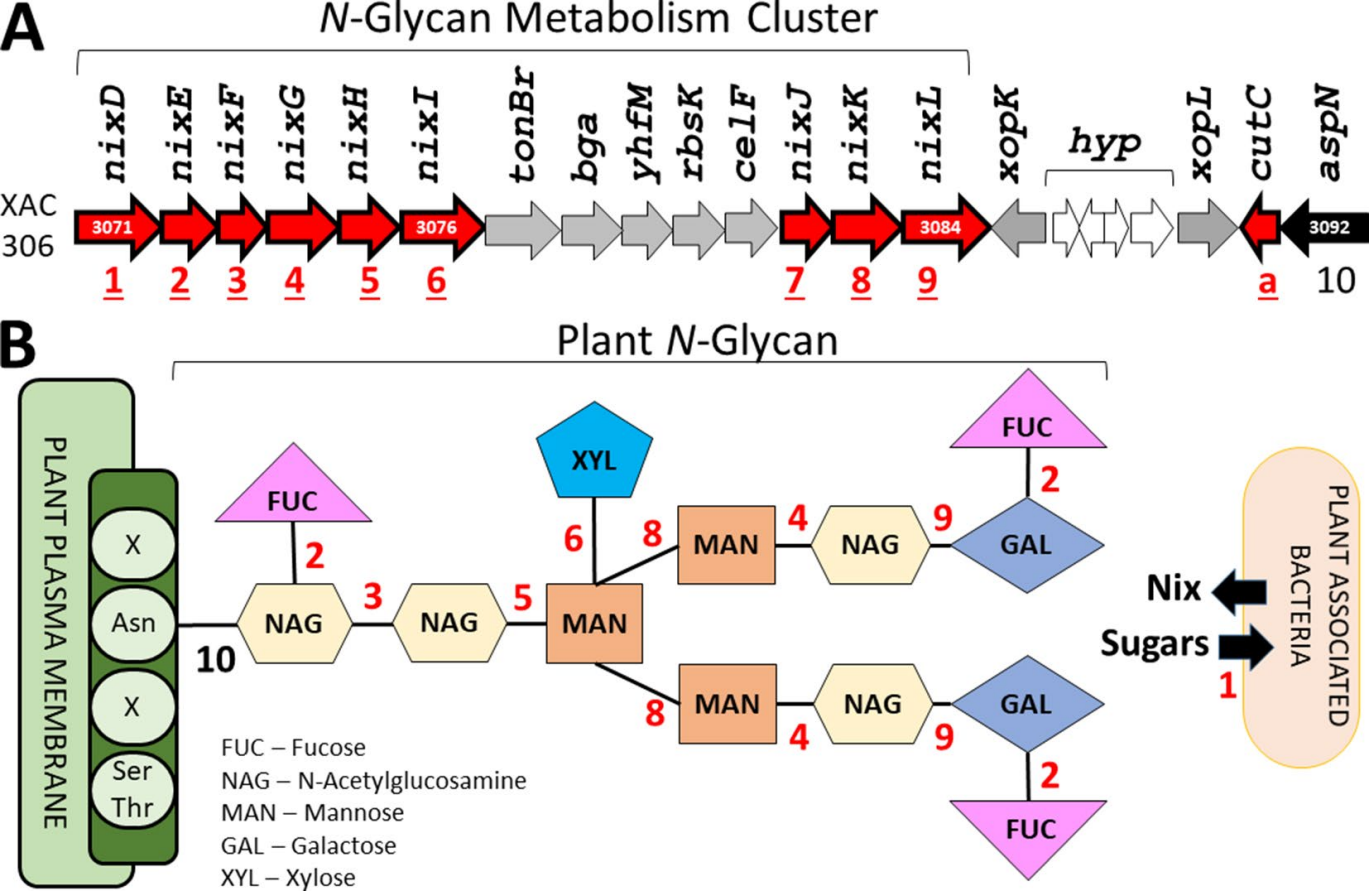
Identification of the genes related with plant N-glycan degradation. **(A)** N-Glycan metabolism gene cluster in Xac306 genome. Red – Genes identified as exclusive of plant-associated genomes. The numbers 1 to 10 identify all genes related to N-glycan degradation. a – Non-related to N-glycan degradation. **(B)** Model of plant N-glycan structure. The numbers 1 to 10 identify the catalytic site of the proteins coded by the genes described in **(A)**. Asn – Asparagine residue. Ser/Thr – Serine and threonine residues. X – Any residue.

Finally, the last protein family unique to plant-associated genome is coded by a TonB-dependent receptor (TBDR) homologous to XAC4131. Blanvillain et al. (2007) predicted 72 TBDR in *Xanthomonas campestris*, several of them belong to carbohydrate-utilization loci involved in the utilization of various plant carbohydrates such as sucrose, plant cell wall compounds and pectin, a major cell wall polymer in plants. Thus, the bacteria may also use the byproducts as energy source by internalizing the monomers through TBDR, an outer membrane protein mainly known for active transport of molecules. Curiously, 10 paralogous of this gene was found at investigated genomes (**Table 2**). One of this paralogous is coded by the gene XAC3071 in Xac306 genome, that corresponds to nixD, the first gene of the nix cluster previously described (**Figure 9A**). It is possible that this TBDR gene are involved with internalization of sugars derivative of N-glycan degradation, which could be used as an alternative source of carbon after suppression of the plant immunity.

This analysis of the repertoire of genes investigated allows us to infer that GTACG tool proved to be efficient in the search for a set of genetic information correlated with a phenotype of interest since the genes identified as unique to plant-associated genomes have already been described as capable of modulating bacterial adaptation to the host plant.

## Conclusions

GTACG is a framework to support the research on bacterial genomes in the area of systems biology, especially the research related to the discovery of genetic knowledge pertaining to the expression of phenotypes. The searches are mainly done using the metrics for the study of pangenomes, such as the number of genomes present in a particular family, but metrics have also been used and developed to express the correlation of families with groups of genomes. GTACG structures information by a top-down model, in which the genomic data and global statistics are first presented to users, followed by the search for families of interest, and then the analysis each family in detail. GTACG encompasses the functionalities already present in some other frameworks on pangenomes, such as the automatic identification of families, identification of core/accessory genome, construction of phylogeny, and visualization of data. However, this framework presents its results in the form of a static website, which makes it easier for users lacking computational knowledge to publish their results and share searches in a simple and efficient way.

## Data Availability

The datasets generated for this study can be found in the GTACG online interface at http://143.107.58.250/reportXantho161.45/. The GTACG is an open source project available at https://github.com/caiorns/GTACG-backend and https://github.com/caiorns/GTACG-frontend.

## Author Contributions

CS and LD designed and implemented the comparative genomics framework. CS, RA, LM and LD selected the strains.CS and LD performed the *in silico* assays. CS, RA, LM and LD analyzed the results and wrote the manuscript. CS, RA, LM and LD revised the manuscript.

## Funding

This work was supported by the following agencies: Saõ Paulo Research Foundation – FAPESP (process 2018/03428-5), and Coordination for the Improvement of Higher Education Personnel – CAPES (the BIGA Project, CFP 51/2013, process 3385/2013). LM has a research fellowship from CNPq. CS has a PhD fellowship from CAPES.

## Conflict of Interest Statement

The authors declare that the research was conducted in the absence of any commercial or financial relationships that could be construed as a potential conflict of interest.
