# Supplementary material for: Gene Tags Assessment by Comparative Genomics (GTACG): A User-Friendly Framework for Bacterial Comparative Genomics
Source: Front Genet. 2019 Aug 26;10:725. doi: 10.3389/fgene.2019.00725 (PMC6718126; doi:10.3389/fgene.2019.00725)
Supplement: Supplementary file 1 [file Table_1.pdf]

Supplementary Material

1 LIST OF GENOMES

PP – Phytopathogenic; P – Phytopathogenic, NP – Non-phytopathogenic.
PA – Plant-associated; P – Plant-associated, Non plant-associated.

Genome Name	Abbreviation	PP	PA	Accession Number	Used in the test with 10 genomes	Used in the test with 20 genomes	Used in the test with 30 genomes	Used in the test with 40 genomes	Used in the test with 50 genomes
Xanthomonas campestris pv. *campestris* str. ATCC 33913	XccATCC33913	P	P	GCA_000007145.1	X	X	X	X	X
Xanthomonas citri pv. *vignicola* CFBP7111	XcvignicolaCFBP7111	P	P	GCA_002218245.1				X	X
Xanthomonas sacchari strain R1	XsacchariR1	NP	P	GCA_000815185.1					
Xanthomonas citri subsp. *citri* strain BL18	XccBL18	P	P	GCA_000961395.1					X
Xanthomonas citri subsp. *citri* LJ207-7	XccLJ207-7	P	P	GCA_001922085.1					X
Xanthomonas oryzae pv. *oryzae* KACC 10331	XooKACC10331	P	P	GCA_000007385.1					
Stenotrophomonas maltophilia AA1	SmaltAA1	NP	NP	GCA_002025605.1					
Xanthomonas citri pv. *phaseoli* var. *fuscans* CFBP6994R	XcpfuscansCFBP6994R	P	P	GCA_002759175.1				X	X
Xylella fastidiosa subsp. sandyi Ann-1	XyfSandyiAnn-1	P	P	GCA_000698805.1					
Xanthomonas citri subsp. *citri* TX160149	XccTX160149	P	P	GCA_002139955.1					
Xanthomonas gardneri JS749-3	XgardneriJS749-3	P	P	GCA_001908755.1					
Pseudoxanthomonas suwonensis strain J1	PsuwonJ1	NP	NP	GCA_000972865.1					
Xanthomonas oryzae pv. *oryzae* PXO99A	XooPXO99A	P	P	GCA_000019585.2					
Xanthomonas citri pv. *glycines* str. 8ra	Xcglycines8ra	P	P	GCA_001854145.2		X	X	X	X
Xanthomonas citri pv. *phaseoli* var. *fuscans* CFBP6990	XcpfuscansCFBP6990	P	P	GCA_002759315.1			X	X	X
Xanthomonas citri subsp. *malvacearum* XcmN1003	XcimalvXcmN1003	P	P	GCA_002224545.1					
Xylella fastidiosa M23	XyfM23	P	P	GCA_000019765.1					
Xanthomonas oryzae pv. *oryzicola* strain L8	XooryzicolaL8	P	P	GCA_001042855.1					

Xanthomonas oryzae pv. *oryzae* PXO282	XooPXO282	P	P	GCA_001746675.1					
Xanthomonas citri pv. *phaseoli* var. *fuscans* CFBP6975	XcpfuscansCFBP6975	P	P	GCA_002759255.1			X	X	X
Xanthomonas citri subsp. *citri* strain NT17	XccNT17	P	P	GCA_000961195.1					
Xanthomonas fragariae PD885	XfragariaePD885	P	P	GCA_900183975.1					
Xanthomonas campestris strain 17	Xcampestris17	PP	PA	GCA_000972745.1		X	X	X	X
Xanthomonas citri subsp. *malvacearum* MS14003	XcimalvMS14003	PP	PA	GCA_002288585.1					
Xanthomonas citri subsp. *citri* strain AW14	XccAW14	PP	PA	GCA_000961455.1					X
Xanthomonas albilineans GPE PC73	XalbGPEPC73	PP	PA	GCA_000087965.1	X	X	X	X	X
Xanthomonas phaseoli pv. *phaseoli* CFBP6164	XppCFBP6164	PP	PA	GCA_002759115.1					
Xanthomonas vesicatoria ATCC 35937 LMG911	XvesicATCCLMG911	PP	PA	GCA_001908725.1					
Xanthomonas fuscans subsp. *aurantifolii* FDC 1609	XfaurantifoliiFDC1609	PP	PA	GCA_001610815.1					
Stenotrophomonas maltophilia strain ISMMS2R	SmaltISMMS2R	NP	NP	GCA_001274675.1					
Stenotrophomonas maltophilia FDAARGOS 325	SmaltFDAARGOS325	NP	NP	GCA_001071475.1					
Xanthomonas citri pv. *phaseoli* var. *fuscans* CFBP6165	XcpfuscansCFBP6165	PP	PA	GCA_002759215.1			X	X	X
Xanthomonas perforans 91-118	Xperforans91-11891-118	PP	PA	GCA_000192045.3					
Xylella fastidiosa U24D	XyfU24D	PP	PA	GCA_001456275.1					
Xanthomonas citri subsp. *citri* Aw12879	XccAw12879	PP	PA	GCA_000349225.1				X	X
Xanthomonas citri subsp. *citri* strain MN10	XccMN10	PP	PA	GCA_000961255.1					
Xanthomonas citri pv. *vignicola* CFBP7112	XcvignicolaCFBP7112	PP	PA	GCA_002218265.1				X	X
Xanthomonas oryzae pv. *oryzae* PXO71	XooPXO71	PP	PA	GCA_001746595.1					
Xanthomonas campestris pv. *raphani* 756C	Xcraphani756C	PP	PA	GCA_000221965.1		X	X	X	X
Xanthomonas oryzae pv. *oryzicola* strain CFBP7331	XooryzicolaCFBP7331	PP	PA	GCA_001042815.1					
Xanthomonas oryzae pv. *oryzae* MAI134	XooMAI134	PP	PA	GCA_002850175.1					
Xanthomonas oryzae pv. *oryzicola* strain BLS279	XooryzicolaBLS279	PP	PA	GCA_001042775.1					
Xanthomonas citri subsp. *citri* strain jx4	Xccjx4	PP	PA	GCA_000961315.1					
Xanthomonas oryzae pv. *oryzae* MAI73	XooMAI73	PP	PA	GCA_002850075.1					
Xanthomonas campestris pv. *campestris* strain B100	XccB100	PP	PA	GCA_000070605.1	X	X	X	X	X
Xylella fastidiosa Pr8x	XyfPr8x	PP	PA	GCA_001456295.1					
Xanthomonas oryzae pv. *oryzae* MAFF 311018 DNA	XooMAFF311018DNA	PP	PA	GCA_000010025.1					
Stenotrophomonas maltophilia K279a strain K279a	SmaltK279a	NP	NP	GCA_000072485.1					

Xanthomonas citri subsp. *citri* LL074-4	XccLL074-4	PP	PA	GCA_001922045.1					X
Xanthomonas oryzae pv. *oryzae* PXO145	XooPXO145	PP	PA	GCA_001746615.1					
Xanthomonas citri subsp. *citri* TX160197	XccTX160197	PP	PA	GCA_002139995.1					
Stenotrophomonas sp. LM091	SLM091LM091	NP	NP	GCA_001806305.1					
Xanthomonas oryzae pv. *oryzae* XF89b	XooXF89b	PP	PA	GCA_002023005.1					
Xylella fastidiosa 9a5c	Xyf9a5c	PP	PA	GCA_000006725.1					
Xanthomonas citri subsp. *citri* strain FB19	XccFB19	PP	PA	GCA_000961375.1					X
Xanthomonas axonopodis Xac29-1	XaxXac29-1	PP	PA	GCA_000348585.1	X	X	X	X	X
Xanthomonas hortorum B07-007	XhortorumB07-007	PP	PA	GCA_002285515.1					
Stenotrophomonas maltophilia R551-3	SmaltR551-3	NP	NP	GCA_000020665.1					
Xanthomonas citri pv. *mangiferaeindicae*	Xcmangifer	PP	PA	GCA_002240395.1		X	X	X	X
Xanthomonas citri pv. *phaseoli* var. *fuscans* CFBP6996R	XcpfuscansCFBP6996R	PP	PA	GCA_002759195.1				X	X
Xanthomonas translucens pv. *translucens* DSM 18974	XttDSM18974	PP	PA	GCA_900094325.1					
Xanthomonas euvesicatoria LMG930	XeuvesicLMG930	PP	PA	GCA_001908795.1					
Xanthomonas fragariae NBC2815	XfragariaeNBC2815	PP	PA	GCA_900183985.1					
Xanthomonas oryzae pv. *oryzae* PXO524	XooPXO524	PP	PA	GCA_001746695.1					
Stenotrophomonas rhizophila QL-P4	SrhizophilaQL-P4	NP	NP	GCA_001704155.1					
Xanthomonas citri pv. *phaseoli* var. *fuscans* CFBP6991	XcpfuscansCFBP6991	PP	PA	GCA_002759395.1			X	X	X
Stenotrophomonas maltophilia CSM2	SmaltCSM2	NP	NP	GCA_002847385.1					
Xylella fastidiosa Hib4	XyfHib4	PP	PA	GCA_001456315.1					
Xanthomonas phaseoli pv. *phaseoli* CFBP6546R	XppCFBP6546R	PP	PA	GCA_002759135.1					
Xanthomonas citri subsp. *citri* strain AW15	XccAW15	PP	PA	GCA_000961475.1					X
Xanthomonas citri pv. *phaseoli* var. fuscans CFBP6988R	XcpfuscansCFBP6988R	PP	PA	GCA_002759275.1			X	X	X
Xylella fastidiosa subsp. *pauca* De Donno	XyfDeDonno	PP	PA	GCA_002117875.1					
Xanthomonas citri subsp. *citri* strain UI7	XccUI7	PP	PA	GCA_000961155.1					
Xanthomonas vesicatoria LM159	XvesicLM159	PP	PA	GCA_001908815.1					
Xanthomonas fuscans subsp. *fuscans* str. 4834-R, chromosome	Xff4834-R	PP	PA	GCA_000969685.1					
Xanthomonas citri subsp. *malvacearum* MSCT	XcimalvMSCT	PP	PA	GCA_001719145.1					
Xanthomonas oryzae pv. *oryzicola* strain RS105	XooryzicolaRS105	PP	PA	GCA_001042875.1					
Xanthomonas citri subsp. *citri* LH201	XccLH201	PP	PA	GCA_001922105.1				X	X
Xanthomonas citri pv. *citri* strain jx-6	Xccitrijx-6	PP	PA	GCA_001028285.3		X	X	X	X

Xylella fastidiosa M12	XyfM12	PP	PA	GCA_000019325.1					
Xanthomonas oryzae pv. *oryzae* PXO83	XooPXO83	PP	PA	GCA_001518895.1					
Xanthomonas citri subsp. *citri* strain MN11	XccMN11	PP	PA	GCA_000961235.1					
PseudoXanthomonas suwonensis 11-1	Psuwon11-1	NP	NP	GCA_000185965.1					
Xanthomonas fuscans subsp. *aurantifolii* 1566	Xfaurantifolii1566	PP	PA	GCA_001610915.1					
Stenotrophomonas maltophilia FDAARGOS 92	SmaltFDAARGOS92	NP	NP	GCA_002951115.1					
Xanthomonas citri pv. *phaseoli* var. *fuscans* CFBP6166	XcpfuscansCFBP6166	PP	PA	GCA_002759235.1			X	X	X
Xanthomonas citri pv. *vignicola* CFBP7113	XcvignicolaCFBP7113	PP	PA	GCA_002218285.1				X	X
Xanthomonas oryzae pv. *oryzicola* strain CFBP7341	XooryzicolaCFBP7341	PP	PA	GCA_001042835.1					
Xanthomonas perforans LH3	XperforansLH3	PP	PA	GCA_001908855.1					
Xanthomonas oryzae pv. *oryzae* MAI106	XooMAI106	PP	PA	GCA_002850135.1					
Xanthomonas citri subsp. *citri* strain 5208	Xcc5208	PP	PA	GCA_000961415.1					X
Stenotrophomonas acidaminiphila strain ZAC14D2 NAIMI4 2	Sacidaminiphila	NP	NP	GCA_001314305.1					
Xanthomonas citri subsp. *citri* UI6	XccUI6	PP	PA	GCA_000961175.1					
Xanthomonas oryzae pv. *oryzae* PXO211	XooPXO211	PP	PA	GCA_001746635.1					
Xanthomonas citri subsp. *citri* strain jx5	Xccjx5	PP	PA	GCA_000961295.1					
Xanthomonas oryzae pv. *oryzae* MAI145	XooMAI145	PP	PA	GCA_002850095.1					
Xylella fastidiosa Temecula1	XyfTemecula1	PP	PA	GCA_000007245.1					
Xanthomonas oryzae pv. *oryzicola* BLS256	XooryzicolaBLS256	PP	PA	GCA_000168315.3					
Stenotrophomonas maltophilia strain ISMMS3	SmaltISMMS3	NP	NP	GCA_001274595.1					
Xanthomonas oryzae pv. *oryzicola* strain BXOR1	XooryzicolaBXOR1	PP	PA	GCA_001042795.1					
Xanthomonas campestris pv. *campestris* strain ICMP 21080	XccICMP21080	PP	PA	GCA_001186415.1		X	X	X	X
Xanthomonas campestris pv. *vesicatoria*	Xcvesicatoria	PP	PA	GCA_000009165.1		X	X	X	X
Xanthomonas oryzae pv. *oryzae* MAI95	XooMAI95	PP	PA	GCA_002850195.1					
Xanthomonas citri subsp. *citri* strain gd2	Xccgd2	PP	PA	GCA_000961355.1					X
Stenotrophomonas maltophilia NCTC10257	SmaltNCTC10257	NP	NP	GCA_900186865.1					
Xanthomonas campestris pv. *campestris* 3811	Xcc3811	PP	PA	GCA_002879955.1	X	X	X	X	X
Xanthomonas campestris pv. *campestris* str. CN12	XccCN12CN12	PP	PA	GCA_002776775.1	X	X	X	X	X
Xanthomonas fragariae	Xfragariae	PP	PA	GCA_001705565.1					
Xanthomonas axonopodis pv. *citri* str. 306	Xaxcitri306	PP	PA	GCA_000007165.1	X	X	X	X	X
Xanthomonas oryzae pv. *oryzae* AXO1947	XooAXO1947	PP	PA	GCA_001466505.1					

2 EXECUTION TIME O SINTETICAL EXPERIMENTS

Xanthomonas oryzae pv. *oryzae* PXO563	XooPXO563	PP	PA	GCA_001746715.1					
Xylella fastidiosa MUL0034	XyfMUL0034	PP	PA	GCA_000698825.1					
Xanthomonas phaseoli pv. *phaseoli* CFBP6982	XppCFBP6982	PP	PA	GCA_002759155.1					
Xanthomonas citri pv. *phaseoli* var. *fuscans* CFBP6992	XcpfuscansCFBP6992	PP	PA	GCA_002759335.1			X	X	X
Xanthomonas citri pv. *phaseoli* var. *fuscans* CFBP7767	XcpfuscansCFBP7767	PP	PA	GCA_002759375.1				X	X
Xanthomonas citri subsp. *citri* strain AW16	XccAW16	PP	PA	GCA_000961495.1					X
Xanthomonas citri subsp. *citri* LH276	XccLH276	PP	PA	GCA_001922065.1				X	X
Xylella fastidiosa 3124	Xyf3124	PP	PA	GCA_001456195.1					
Stenotrophomonas sp. *WZN-1*	SWZN-1WZN-1	NP	NP	GCA_002192255.1					
Xanthomonas translucens pv. *undulosa* strain Xtu 4699	XtuXtu4699	PP	PA	GCA_001021935.1					
Stenotrophomonas maltophilia strain ISMMS2	SmaltISMMS2	NP	NP	GCA_001274655.1					
Xanthomonas citri pv. *phaseoli* var. *fuscans* CFBP6989	XcpfuscansCFBP6989	PP	PA				X	X	X
Xanthomonas citri subsp. *citri* TX160042	XccTX160042	PP	PA	GCA_002139975.1					
Xanthomonas fragariae PD5205	XfragariaePD5205	PP	PA	GCA_900183995.1					
Xanthomonas oryzae pv. *oryzae* PXO86	XooPXO86	PP	PA	GCA_000948075.1					
Xanthomonas citri subsp. *malvacearum* XcmH1005	XcimalvXcmH1005	PP	PA	GCA_002224525.1					
Xanthomonas citri pv. *glycines* str. 12-2	Xcglycines12-2	PP	PA	GCA_002163775.1		X	X	X	X
Stenotrophomonas maltophilia D457	SmaltD457	NP	NP	GCA_000284595.1					
Xanthomonas gardneri ICMP7383	XgardneriICMP7383	PP	PA	GCA_001908775.1					
Xanthomonas oryzae pv. *oryzicola* strain YM15	XooryzicolaYM15	PP	PA	GCA_001021915.1					
Xanthomonas citri subsp. *citri* strain MN12	XccMN12	PP	PA	GCA_000961215.1					
Xanthomonas citri pv. *phaseoli* var. *fuscans* CFBP6167	XcpfuscansCFBP6167	PP	PA	GCA_002759415.1			X	X	X
Xanthomonas oryzae pv. *oryzae* PXO236	XooPXO236	PP	PA	GCA_001746655.1					
Xylella fastidiosa subsp. *fastidiosa* GB514	XyffGB514	PP	PA	GCA_000148405.1					
Xanthomonas oryzae pv. *oryzicola* strain CFBP7342	XooryzicolaCFBP7342	PP	PA	GCA_000940825.1					
Xanthomonas phaseoli pv. *phaseoli* CFBP412	XppCFBP412	PP	PA	GCA_002759095.1					
Xanthomonas citri pv. *phaseoli* var. *fuscans* CFBP4885	XcpfuscansCFBP4885	PP	PA	GCA_002759355.1			X	X	X
Xanthomonas citri subsp. *citri* strain AW13	XccAW13	PP	PA	GCA_000961435.1					X

Xanthomonas fuscans subsp. *aurantifolii* FDC 1559	XfaurantifoliiFDC1559	PP	PA	GCA_001610795.1					
Stenotrophomonas maltophilia JV3	SmaltJV3	NP	NP	GCA_000223885.1					
Stenotrophomonas sp. KCTC 12332 YM1	SKCTC12332YM1	NP	NP	GCA_001562215.1					
Xanthomonas campestris pv. *vesicatoria* str. 85-10	Xcvesicatoria85-10	PP	PA	GCA_000009165.1		X	X	X	X
Xylella fastidiosa Fb7	XyfFb7	PP	PA	GCA_001456335.3					
Xanthomonas citri subsp. *citri* A306	XccA306	PP	PA	GCA_000816885.1				X	X
Xanthomonas citri subsp. *malvacearum* AR81009	XcimalvAR81009	PP	PA	GCA_002288565.1					
Xanthomonas citri subsp. *citri* strain mf20	Xccmf20	PP	PA	GCA_000961275.1					
Xanthomonas oryzae pv. *oryzae* MAI129	XooMAI129	PP	PA	GCA_002850155.1					
Xanthomonas oryzae pv. *oryzicola* strain B8-12	XooryzicolaB8-12	PP	PA	GCA_001042745.1					
Xanthomonas oryzae pv. *oryzae* MAI68	XooMAI68	PP	PA	GCA_002850115.1					
Xanthomonas citri subsp. *citri* strain gd3	Xccgd3	PP	PA	GCA_002759095.1					
Xanthomonas oryzae pv. *oryzicola* strain CFBP2286	XooryzicolaCFBP2286	PP	PA	GCA_001042735.1					
Xanthomonas oryzae pv. *oryzae* PXO602	XooPXO602	PP	PA	GCA_001746735.1					
Xanthomonas campestris pv. *campestris* strain ICMP 4013	XccICMP4013	PP	PA	GCA_001186465.1		X	X	X	X
Xanthomonas oryzae pv. *oryzae* MAI99	XooMAI99	PP	PA	GCA_002850215.1					
Xanthomonas campestris pv. *campestris* str. CN18	XccCN18CN18	PP	PA	GCA_002776835.1	X	X	X	X	X
Xanthomonas campestris pv. *campestris* str. 8004	Xcc8004	PP	PA	GCA_000012105.1	X	X	X	X	X
Stenotrophomonas maltophilia OUC Est10	SmaltOUCEst10	NP	NP	GCA_002138415.1					
Stenotrophomonas nitritireducens 2001	Snitrit2001	NP	NP	GCA_001700965.1					
Xylella fastidiosa J1a12	XyfJ1a12	PP	PA	GCA_001456235.1					
Xanthomonas axonopodis pv. *citrumelo* F1	XaxcitrumeloF1	PP	PA	GCA_000225915.1	X	X	X	X	X
PseudoXanthomonas spadix BD-a59	PspadixBD-a59	NP	NP	GCA_000233915.4					

Table S1. List of genomes. A total of 161 genomes from the Xanthomonadaceae family, obtained from the NCBI database, presented to the GTACG framework to a comparative study to find correlated genes which explain the association of some bacteria with plants.

		Genomes				
	# of Threads	10	20	30	40	50
List all sequences	–	4.04	6.87	9.68	12.59	16.14
Make Blast	5	2497.39	10183.3	23178.64	42025.58	66661.24
	10	1289.03	5110.52	11751.47	21296.11	33769.58
	15	1023.38	3921.07	9419.43	16392.82	26008.75
	20	885.14	3703.94	8775.91	15648.00	25039.39
Make MMseqs2	5	427.51	1054.93	1875.99	2863.77	4010.84
	10	221.43	548.52	969.83	1480.42	2070.17
	715	180.64	445.35	786.23	1203.90	1678.29
	20	171.60	419.92	742.24	1131.78	1591.18
Sequence Clustering	1	31.05	190.48	903.73	2349.09	5555.40
	5	17.97	77.23	288.24	674.20	1567.47
	10	17.14	68.39	236.13	511.04	1140.76
	15	16.68	63.22	207.29	438.43	977.97
	20	17.63	61.04	210.34	417.69	929.38
Cluster as Graph	–	16.13	58.41	139.76	309.14	527.36
Make alignments and trees	1	2030.26	5287.93	7399.21	11281.53	12476.08
	5	429.53	1127.85	1576.07	2425.43	2674.81
	10	250.71	617.00	848.27	1413.39	1536.44
	15	204.48	498.76	750.32	1214.65	1321.51
	20	196.08	453.62	664.70	1110.95	1231.06
Export results as a report	1	79.04	221.93	355.99	818.63	926.91
	5	36.63	102.83	194.98	402.33	476.25
	10	37.09	99.71	186.99	374.31	460.37
	15	35.94	95.37	178.98	361.77	449.08
	20	33.50	91.64	177.46	322.47	455.82

Table S2. Execution time for sintetical experimets with 10, 20, 30, 40 and 50 genomes. All runs were made on an Intel(R) Xeon(R) CPU E5-2620 with 24 core and the times are presented in seconds.
